# Understanding the development of depression through emotion beliefs, emotion regulation, and parental socialisation

**DOI:** 10.1038/s41598-025-08758-w

**Published:** 2025-07-15

**Authors:** Susanne Peter, Olivia S. Riddle, Bonamy R. Oliver, Matthew P. Somerville

**Affiliations:** IOE, UCL’s Faculty of Education and Society, London, WC1H 0AL UK

**Keywords:** Human behaviour, Psychology

## Abstract

The capacity to understand and regulate emotions flexibly and productively is a key driver of mental health and well-being. Parents’/carers’ role in emotion socialisation—specifically, their response to children’s expressions of emotion—has significant influence on the development of these processes. We examined emotion controllability beliefs (ECBs) and cognitive reappraisal as underlying mechanisms of the relationship between recalled parental emotion responses and depression across two studies. In Study 1 (*N* = 215, 65.6% female, *M*_age_ = 29.10), we found that supportive parental responses were associated with lower levels of depression via increased use of cognitive reappraisal. Study 2 (*N* = 410, 51.7% female, *M*_age_ = 46.24) replicated the findings of Study 1 by repeating the analysis using a larger sample, more representative of the UK population in terms of age, gender, and ethnicity. Beyond confirming the reappraisal pathway, Study 2 also revealed that ECBs mediated these relationships, with supportive parental responses predicating stronger beliefs in the controllability of emotions, subsequently predicting greater reappraisal use and lower depression. These findings extend our understanding of the mechanisms linking early emotion socialisation to later mental health outcomes while highlighting the importance of supportive parental responses for long-term outcomes.

## Introduction

Mental health problems are a global public health concern, including behavioural disorders such as antisocial and conduct disorders, and emotional disorders such as anxiety and depression. Depression is one of the most prevalent of these disorders^1^, and has been consistently linked with poorer life outcomes, greatly impacting the professional, educational and social functioning of those who experience it, as well as being associated with suicide, currently the leading cause of death for 15- to 29-year-olds^1^. Importantly, symptoms of depression that do not reach diagnostic threshold are not uncommon in community and convenience samples (e.g., undergraduate students), and are considered to impact quality of life, not least since they convey an increased risk of major depression over the life span^2^. Around half of all adult mental-health disorders appear before age 18^3^ such that understanding the developmental psychological processes contributing to their onset and persistence is an essential area of enquiry. For depression, a disorder of emotion, considerable research attention has considered the role of parent/carer emotion socialisation, that is how a parent responds to their children’s emotions, discusses emotions, and expresses their own emotions^4^. This socialisation is seen to play a central role in children’s psychological well-being, lowering the risk of depression in the short^5^ and long^6^ term. Yet, the mechanisms through which these parental influences exert their effects are not well understood, particularly in terms of parental responses to emotional expression. In the current study, we examined emotion beliefs and a well-understood aspect of emotion regulation, cognitive reappraisal, as potential mechanisms through which parental emotion responses in childhood contribute to depression outcomes in adulthood.

Parent emotion socialisation is posited to shape how children come to understand, experience, express and regulate their emotions when they arise^7,8^. This socialisation may involve modelling, whereby parents demonstrate through their own values, beliefs, and practices—for example, staying calm during a stressful situation by taking deep breaths to regulate their own emotions^7^. Emotion coaching or direct teaching of emotional expression may also be used to soothe or validate difficult feelings, encouraging emotional expression in a socially acceptable way^9,10^. For instance, a parent might listen to and acknowledge a child’s feelings and encourage them to articulate their emotions with words by saying, “I can see that you are feeling sad because your friend couldn’t come over. It’s okay to feel sad; I would feel sad too.” In contrast to these supportive emotional responses, parents may be emotionally unsupportive, for example minimising, criticising, or punishing emotional expression, or becoming inappropriately distressed themselves. These unsupportive parental emotion responses have been shown to negatively relate to children’s emotional competence and mental health^4,11–13^. Importantly, evidence suggests that early parental emotion responses may persist and lay the foundation for adult mental health. For example, a study which explored the relationship between remembered parental emotion socialisation found that non-supportive emotion practices in childhood predicted higher levels of anxiety in adulthood^14^. Furthermore, punitive responses exhibited by parents have been shown to predict higher levels of internalising symptoms in adolescence and young adulthood^15,16^. The mechanisms and processes linking these parental responses to children’s emotion and mental health outcomes are not well understood. One potential candidate is emotion regulation, due to the strong link between parenting practices and the development of emotion regulation^7^.

Emotion regulation is defined as both effortful and automatic processes that influence the expression of an emotional response in terms of occurrence, magnitude, and duration^17^, and is used to monitor and alter emotional experiences and expressions^7^. The underlying processes of emotion regulation involve identifying the need to regulate, the selection and implementation of an emotion regulation strategy and monitoring of its success^17^. The origin of emotion regulation is likely to be complex, but parents are thought to have a key role, and findings suggest that parental emotion responses may be important. For example, mothers’ minimising and punitive reactions to negative emotions have been shown to relate to children’s tendency to regulate their emotional response by escaping rather than expressing their negative emotions^18^. One adaptive emotion regulation strategy is cognitive reappraisal, whereby a person seeks to alter their thoughts about a situation in order to modify its emotional significance^17^. Cognitive reappraisal is one of the best understood emotion regulation strategies and has been linked with diverse mental health outcomes, such as high levels of life satisfaction, optimism, as well as lower levels of anxiety and depression^19–21^. Research focusing on parental socialisation and specific emotion regulation strategies has established links between supportive parenting and increased use of reappraisal in children^22,23^. Several mechanisms may explain this connection: parents who model reappraisal provide observational learning opportunities; parent–child emotional conversations help children develop vocabulary for reframing experiences; and supportive reactions create a safe environment for practicing perspective taking. Conversely, non-supportive reactions that devalue emotions may promote avoidance and suppression in children, inhibiting active cognitive engagement. These relationships remain significant even in studies using retrospective assessment of parental reactions^23^. Thus, we suggest that cognitive reappraisal may be a good candidate for better understanding the processes by which parental emotion responses associate with mental health outcomes.

Importantly, little is known about why, whether and when emotion regulation strategies are employed^24^. This understanding may be important for knowing how to help individuals improve these strategies and thus ultimately reduce mental health difficulties associated with poor emotion regulation, including depression. Recent evidence suggests that the implicit beliefs individuals hold about the degree to which they can control their emotions (emotion controllability beliefs; ECBs) may relate to the likelihood that they engage in emotion regulation^25^. In short, ECBs are considered to influence the extent to which individuals are motivated to engage in emotion regulation – a position compellingly supported by evidence from recent systematic reviews^26,27^– and that this in turn relates to the development of depression^28^. Initial evidence suggests that individuals who perceive emotions to be uncontrollable report more negative emotion and greater depression than those who perceive emotion as within their control^24,29,30^, and those who believe emotions can be controlled show greater wellbeing^31^. It is posited that those who believe emotions can be controlled benefit from higher levels of emotion regulation self-efficacy, increasing their motivation to attempt emotion regulation and to sustain these attempts^29,30,32^. Despite their importance, little is known about the origins of ECBs.

It is possible that bottom-up processes are responsible for the generation of beliefs. For example, individuals who experience intense emotions or struggle to regulate them may conclude that there is very little they – or anyone – can do to control emotions. Conversely, individuals who find emotional experiences easier to manage or are more successful at regulating their emotions may have more belief in emotions as controllable. Yet, studies examining bidirectional links between ECBs, emotion regulation and depression have found stronger support for beliefs predicting emotion regulation and depression, than depression or emotion regulation predicting beliefs^33,34^. Another possibility is that ECBs are generated from the top down. For example, children’s beliefs may be influenced by those around them, such as peers or family members, through observation, socialisation, or explicit messages focused on the expression or regulation of emotions^35^. We argue that parents, and parental emotion responses in particular, may play an important role in shaping these beliefs. This understudied area forms the theoretical foundation for our study.

In two samples of adults reporting retrospectively on parental emotion responses in childhood, the current study predicted that: independent of ECBs, cognitive reappraisal would mediate the relationship between remembered parental emotion responses in childhood and current depression (Hypothesis 1); independent of cognitive reappraisal, ECBs would mediate the relationship between parental emotion responses and depression (Hypothesis 2); and ECBs and cognitive reappraisal would together play a serial mediating role in the relationship between parental emotion responses and depression (Hypothesis 3).

## Study 1

In Study 1 (N = 215), we assessed the relationship between recalled parental emotion responses and depression via cognitive reappraisal and emotion controllability beliefs.

## Results

### Preliminary analyses

Descriptive analyses for all variables are provided in Table 1, along with Pearson’s product-moment correlations between beliefs about emotion controllability, cognitive reappraisal, symptoms of depression, and emotionally supportive and non-supportive parental emotion response practices. Significant relationships between constructs were in expected directions, with the exception of non-supportive parental emotion responses which were not associated with cognitive reappraisal (*p* = 0.127) and ECBs which were not significantly associated with supportive (*p* = 0.085), or non-supportive parental emotion responses (*p* = 0.404).

**Table 1:** Descriptive statistics, cronbach’s alpha, and correlations among all study variables.

Variables	M	SD	Range	a	1	2	3	4	5
1. Emotion controllability beliefs	4.72	1.14	2.00–7.00	0.73	–	0.32***	− 0.23**	0.12	− 0.06
2. Cognitive eappraisal	4.97	1.03	1.83–7.00	0.81		–	− 0.23**	0.23**	− 0.11
3. Depression	1.32	1.09	0.00–4.57	0.87			–	− 0.27***	0.33***
4. Supportive parental emotion responses	4.74	1.21	1.22–7.00	0.95				–	− 0.61***
5. Non − supportive parental emotion responses	2.72	1.06	1.00–5.89	0.92					–

M Mean, SD Standard deviation, a Cronbach’s alpha, *p < 0.05, *p < 0.01, ***p < 0.001.

### Individual mediation analysis

**Fig. 1:**
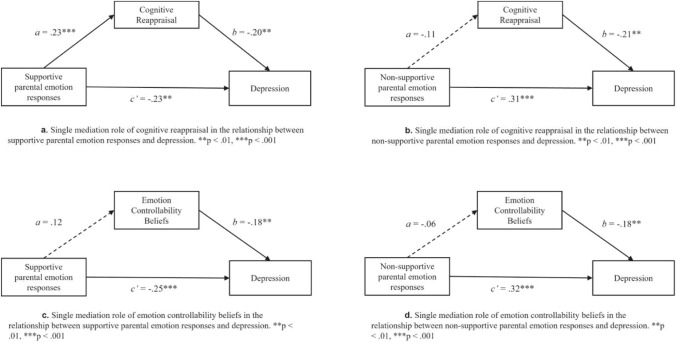
Overview of individual mediation analysis.

Initially, four individual mediation analyses were conducted. First, testing Hypothesis 1, we assessed the indirect effect of supportive (Fig. 1a) and non-supportive (Fig. 1b) parental emotion responses on depression via cognitive reappraisal. Our findings partially supported our hypothesis. That is, the indirect effect of supportive parental emotion responses on depression via cognitive reappraisal (Fig. 1a) was significant (ab = − 0.04, 95% bootstrap CI − 0.09 to − 0.01), with supportive parental emotion responses positively predicting cognitive reappraisal (95% CI − 0.08 to 0.31), negatively predicting depression (95% CI − 0.32 to − 0.08), and cognitive reappraisal negatively predicting depression (95% CI − 0.34 to − 0.06). However, in contrast to expectations, the indirect effect of non-supportive parental emotion responses on depression via cognitive reappraisal was not significant (ab = 0.02, 95% bootstrap CI − 0.01 to 0.06), with non-supportive parental emotion responses positively predicting depression (95% CI 0.19 to 0.44), cognitive reappraisal negatively predicting depression, (95% CI − 0.35 to − 0.09), but non-supportive parental emotion responses not significantly predicting cognitive reappraisal (95% CI − 0.24 to 0.03).

Second, testing Hypothesis 2, we assessed the indirect effect of supportive (Fig. 1c) and non-supportive (Fig. 1d) parental emotion responses on depression via ECBs. Our findings did not support our hypothesis. Specifically, the indirect effect of supportive parental emotion responses via ECBs was not significant (ab = − 0.02, 95% bootstrap CI − 0.06 to 0.00), with supportive parental emotion responses (95% CI − 0.34 to − 0.10) and ECBs (95% CI − 0.29 to − 0.04) negatively predicting depression, but supportive parental emotion responses not predicting ECBs (95% CI − 0.02 to 0.24). Similarly, the indirect effect of non-supportive parental emotion responses via ECBs was not significant (ab = 0.01, 95% CI − 0.02 to 0.04), with non-supportive parental emotion responses positively predicting depression (95% CI 0.20 to 0.45), emotion beliefs negatively predicting depression (95% CI − 0.29 to − 0.05), but non-supportive parental emotion responses not predicting ECBs (95% CI − 0.21 to 0.08).

### Serial mediation analysis

To address Hypothesis 3, a serial mediation analysis was conducted to test the relationship between parental emotion responses and depression as mediated by ECBs and cognitive reappraisal (see Table 2).

**Table 2:** Regression coefficients in the serial mediation analysis.

Path	B	95%CI		t	*p*	Beta
		Lower	Upper			
Depression ← Supportive parental emotion responses	− 0.24	− 0.36	− 0.12	− 4.04	0.000	− 0.27
Depression ← Non-supportive parental emotion responses	0.34	0.21	0.48	5.09	0.000	0.33
Depression ← Emotion Controllability Beliefs	− 0.22	− 0.35	− 0.09	− 3.44	0.001	− 0.23
Depression ← Cognitive Reappraisal	− 0.24	− 0.38	− 0.10	− 3.39	0.001	− 0.23
Cognitive Reappraisal ← Emotion Controllability Beliefs	0.29	0.17	0.41	4.86	0.000	0.32
Cognitive Reappraisal ← Supportive parental emotion responses	0.19	0.08	0.31	3.37	0.001	0.23
Cognitive Reappraisal ← Non-supportive parental emotion responses	− 0.10	− 0.24	0.03	− 1.53	0.127	− 0.11
Emotion Controllability Beliefs ← Supportive parental emotion responses	0.11	− 0.02	0.24	1.7	0.085	0.12
Emotion Controllability Beliefs ← Non-supportive parental emotion responses	− 0.06	− 0.21	0.09	− 0.84	0.404	− 0.06

**Fig. 2:**
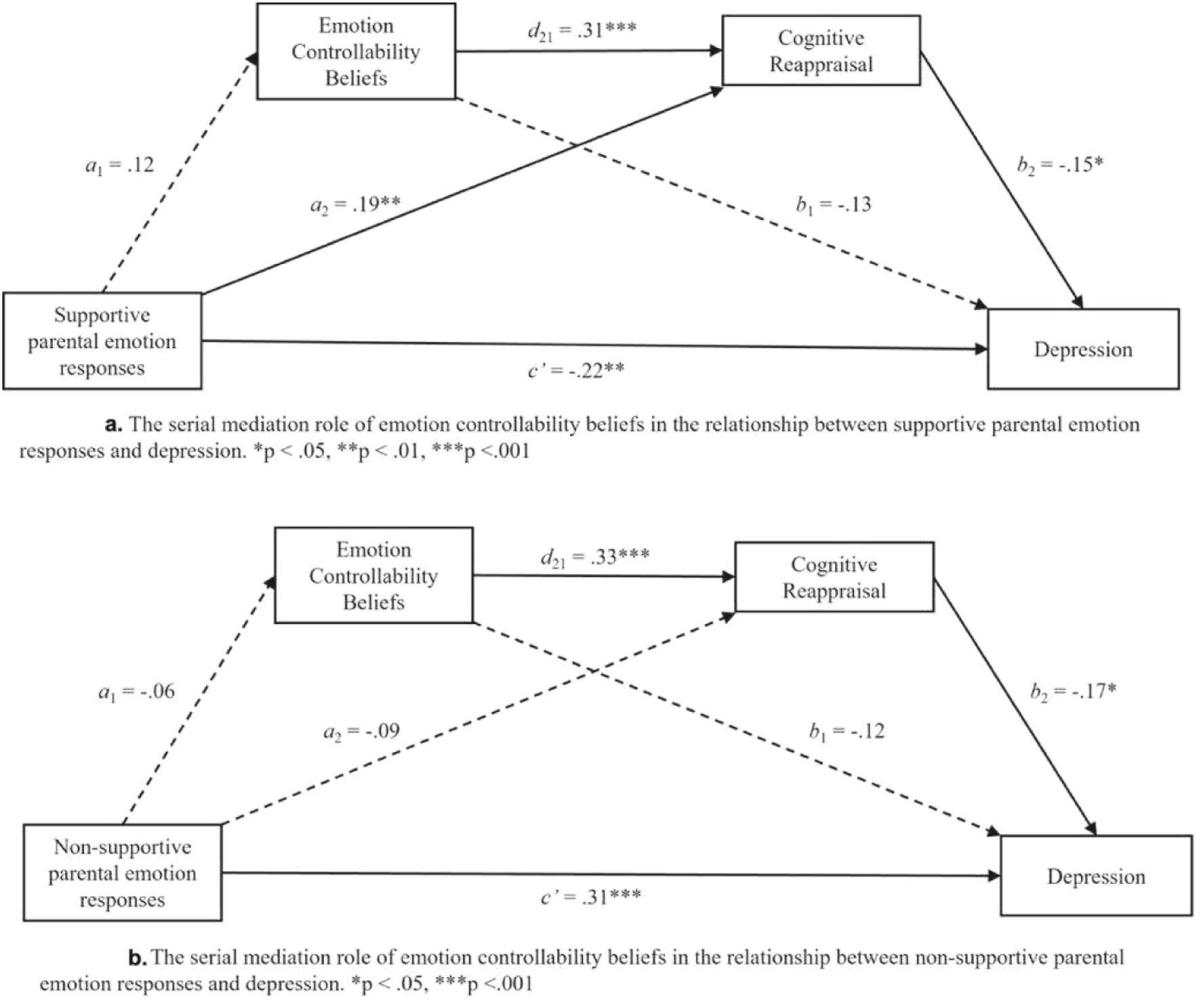
Overview of serial mediation analysis.

In contrast with Hypothesis 3, serial mediation results suggested that despite the full model being significant, F_(1, 208)_ = 16.30, *p* < 0.001, R^2^ = 0.07, the indirect effect of supportive parental emotion responses through the mediators ECBs and cognitive reappraisal was not significant (see Table 3 and Fig. 2a). As foreshadowed by the individual mediation model results, supportive parental emotion responses negatively predicted depression, and positively predicted cognitive reappraisal, but did not predict ECBs.

**Table 3:** Total, direct, and indirect effects of Model 1 (supportive parental emotion responses) and Model 2 (non-supportive parental emotion responses). Based on 5,000 bootstrap samples; 95% Bias corrected confidence interval.

Effect	Estimate	SE	95% CI		t	*p*
			Lower	Upper		
Model 1						
Total effects	− 0.24	0.06	− 0.36	− 0.12	− 4.04	0.000
Direct effect	− 0.19	0.06	− 0.31	− 0.08	− 3.27	0.001
Total indirect effect	− 0.04	0.02	− 0.09	− 0.01	–	–
Indirect effect (X→M1→Y)	− 0.01	0.01	− 0.05	0.00	–	–
Indirect effect (X→M2→Y)	− 0.03	0.02	− 0.06	− 0.00	–	–
Indirect effect (X→M1→M2→Y)	− 0.01	0.00	− 0.02	0.00	–	–
Model 2						
Total effects	0.34	0.07	0.21	0.47	5.09	0.000
Direct effect	0.31	0.06	0.18	0.44	4.8	0.000
Total indirect effect	0.03	0.02	− 0.01	0.07	–	–
Indirect effect (X→M1→Y)	0.01	0.01	− 0.01	0.04	–	–
Indirect effect (X→M2→Y)	0.02	0.01	− 0.02	0.05	–	–
Indirect effect (X→M1→M2→Y)	0.00	0.01	− 0.01	0.01	–	–

Similarly, also in contrast with expectations, serial mediation results suggested that despite the full model being significant (F_(1, 207)_ = 25.86, *p* < 0.001, R^2^ = 0.11), the indirect effect of non-supportive parental emotion responses on depression through the mediators ECBs and cognitive reappraisal was not significant (see Table 3 and Fig. 2b). Non-supportive parental emotion responses positively predicted depression, but did not predict cognitive reappraisal, or ECBs. Moreover, ECBs and cognitive reappraisal both predicted depression negatively, and ECBs positively predicted cognitive reappraisal.

## Study 2

The aim of Study 2 (N = 410) was to replicate the findings of Study 1 by repeating the analysis using a larger sample, more representative of the UK population in terms of age, gender, and ethnicity. Measures and design of the study was the same as in Study 1.

## Results

### Preliminary analysis

Descriptive analyses for all variables are provided in Table 4, along with Pearson’s product-moment correlations between beliefs about emotion controllability, cognitive reappraisal, symptoms of depression, and emotionally supportive and non-supportive parental emotion response practices. Significant relationships between constructs were in expected directions. In contrast to Study 1, Study 2 identifies significant relationships between non-supportive parental responses and cognitive reappraisal (*p* = 0.009). Moreover, ECBs were significantly associated with supportive (*p* = 0.001) and non-supportive parental emotion responses (*p* = 0.042).

**Table 4:** Descriptive statistics, cronbach’s alpha, and correlations among all study variables.

Variables	M	SD	Range	a	1	2	3	4	5
1. Emotion Controllability Beliefs	4.47	1.21	1.00–7.00	0.83	–	0.44***	− 0.22***	0.16**	− 0.10*
2. Cognitive Reappraisal	4.98	0.98	1.00–7.00	0.88		–	− 0.31***	0.29***	− 0.13**
3. Depression	0.83	0.71	0.00–3.00	0.93			–	− 0.15**	0.24***
4. Supportive parental emotion responses	4.14	1.40	1.00–7.00	0.97				–	− 0.46***
5. Non-supportive parental emotion responses	2.94	1.27	1.00–7.00	0.94					–

M Mean, SD Standard deviation, a Cronbach’s alpha, *p < .0.05, *p < 0.01, ***p <0.001.

### Individual mediation analysis

**Fig. 3:**
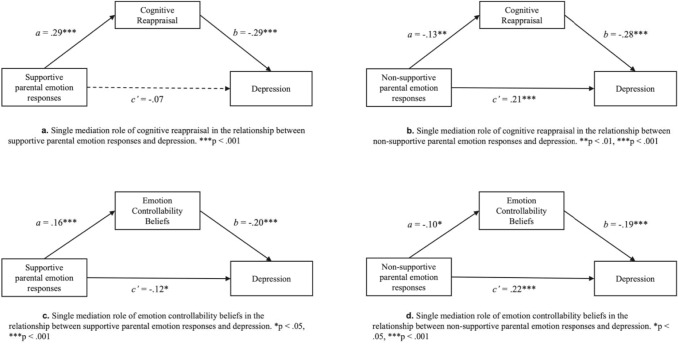
Overview of individual mediation analysis.

Initially, four individual mediation analyses were conducted. First, testing Hypothesis 1, we assessed the indirect effect of supportive (Fig. 3a) and non-supportive (Fig. 3b) parental emotion responses on depression via cognitive reappraisal. Our findings partially supported our hypothesis. The indirect effect of supportive parental emotion responses on depression via cognitive reappraisal (Fig. 3a) was significant (ab = − 0.08, 95% bootstrap CI − 0.13 to − 0.05), with supportive parental emotion responses positively predicting cognitive reappraisal (95% CI 0.14 to 0.27), and cognitive reappraisal negatively predicting depression (95% CI − 0.28 to − 0.14). However, the direct effect of supportive parental emotion responses on depression (95% CI − 0.08 to 0.02) was not significant. Similarly, the indirect effect of non-supportive parental emotion responses on depression via cognitive reappraisal was also significant (ab = 0.04, 95% bootstrap CI 0.01 to 0.07), with non-supportive parental emotion responses positively predicting depression (95% CI 0.06 to 0.17), negatively predicting cognitive reappraisal (95% CI − 0.17 to − 0.02), and cognitive reappraisal negatively predicting depression (95% CI − 0.27 to − 0.14), consistent with Hypothesis 1.

Second, testing Hypothesis 2, we assessed the indirect effect of supportive (Fig. 3c) and non-supportive (Fig. 3d) parental emotion responses on depression via ECBs. Our findings did support our hypothesis for both types of parental responses. That is, the indirect effect of supportive parental emotion responses via ECBs was significant (ab = − 0.03, 95% bootstrap CI − 0.06 to − 0.01), with supportive parental emotion responses (95% CI − 0.11 to − 0.01) and ECBs (95% CI − 0.17 to − 0.06) negatively predicting depression, and supportive parental emotion responses positively predicting ECBs (95% CI 0.05 to 0.22). Similarly, the indirect effect of non-supportive parental emotion responses via ECBs was significant (ab = 0.02, 95% CI 0.00 to 0.04), with non-supportive parental emotion responses positively predicting depression (95% CI 0.07 to 0.18), negatively predicting ECBs (95% CI − 0.19 to 0.00)., and ECBs negatively predicting depression (95% CI − 0.17 to − 0.06).

### Serial mediation analysis

To address Hypothesis 3, a serial mediation analysis was conducted to test the relationship between parental emotion responses and depression as mediated by ECBs and cognitive reappraisal (see Table 5).

**Table 5:** Regression coefficients in the serial mediation analysis.

Path	B	95% CI		t	*p*	Beta
		Lower	Upper			
Depression ← Supportive parental emotion responses	− 0.08	− 0.12	− 0.03	− 3.056	0.000	− 0.15
Depression ← Non-supportive parental emotion responses	0.14	0.08	0.19	5.03	0.000	0.24
Depression ← Emotion Controllability Beliefs	− 0.13	− 0.35	− 0.09	− 4.50	0.000	− 0.22
Depression ← Cognitive Reappraisal	− 0.24	− 0.18	− 0.07	− 3.39	0.000	− 0.23
Cognitive Reappraisal ← Emotion Controllability Beliefs	0.36	0.29	0.43	9.87	0.000	0.44
Cognitive Reappraisal ← Supportive parental emotion responses	0.21	0.14	0.27	6.23	0.000	0.29
Cognitive Reappraisal ← Non-supportive parental emotion responses	− 0.10	− 0.17	0.02	− 2.62	0.01	− 0.13
Emotion Controllability Beliefs ← Supportive parental emotion responses	0.14	0.05	0.22	3.22	0.000	0.16
Emotion Controllability Beliefs ← Non-supportive parental emotion responses	− 0.10	− 0.19	0.00	− 2.04	0.04	− 0.10

**Fig. 4:**
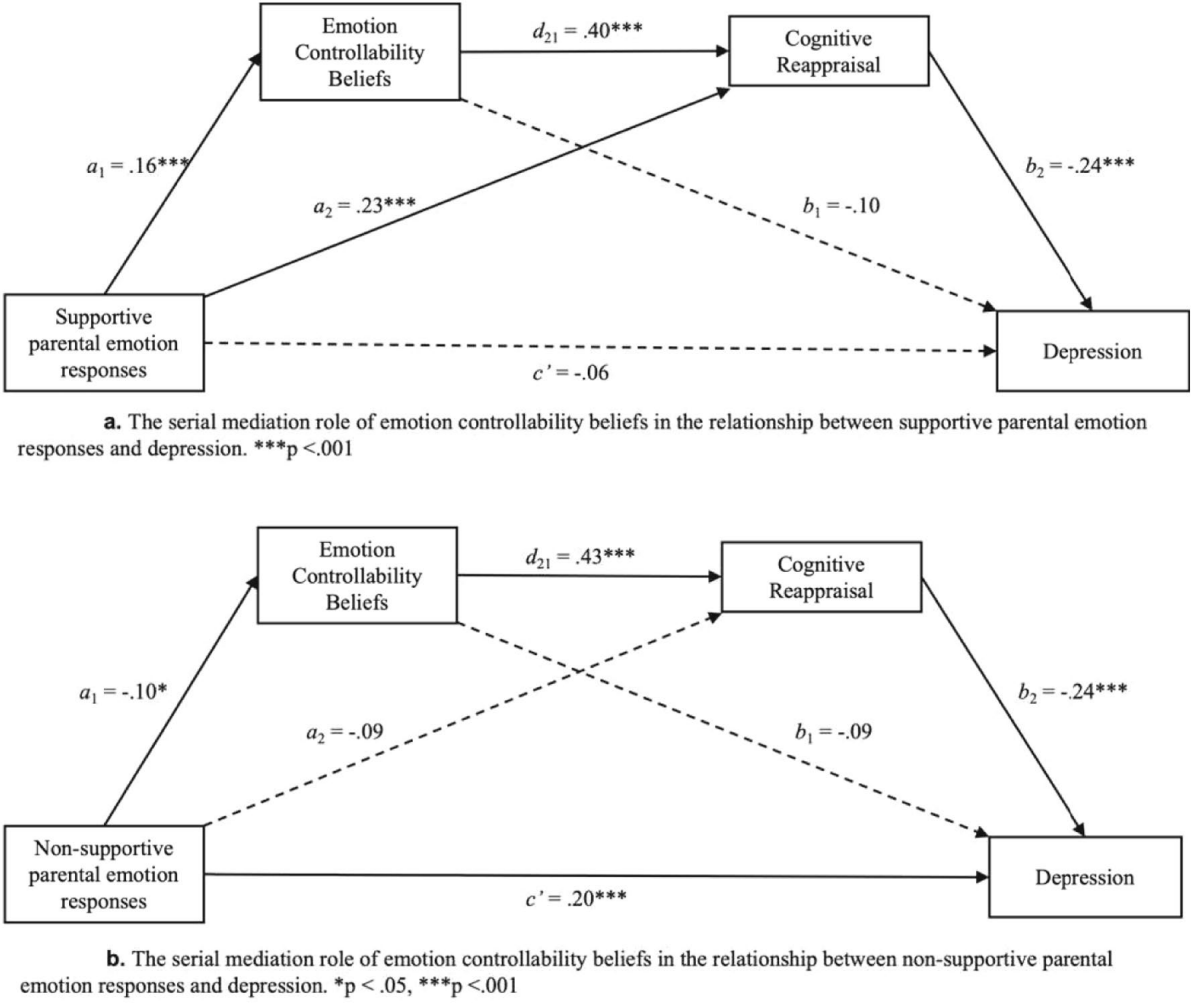
Overview of serial mediation analysis.

In line with our predictions, the serial mediation results for supportive parental emotion responses revealed a significant full model, F (1, 408) = 9.37, *p* < 0.001, R^2^ = 0.02. The indirect effect of supportive parental emotion responses on depression through the sequential mediators of ECBs and cognitive reappraisal was significant (see Table 6 and Fig. 4a). Consistent with the individual mediation model results, supportive parental emotion responses did not predict depression, but positively predicted ECBs and cognitive reappraisal.

**Table 6:** Total, direct, and indirect effects of Model 3 (supportive parental emotion responses) and Model 4 (non-supportive parental emotion responses). Based on 5,000 bootstrap samples; 95% Bias corrected confidence interval.

Effect	Estimate	SE	95%CI		t	*p*
			Lower	Upper		
Model 1						
Total effects	− 0.08	0.02	− 0.12	− 0.03	− 3.06	0.000
Direct effect	− 0.03	0.02	− 0.08	0.02	− 1.26	0.21
Total indirect effect	− 0.09	0.02	− 0.13	− 0.05	–	–
Indirect effect (X→M1→Y)	− 0.02	0.01	− 0.04	0.00	–	–
Indirect effect (X→M2→Y)	− 0.06	0.02	− 0.09	− 0.03	–	–
Indirect effect (X→M1→M2→Y)	− 0.02	0.01	− 0.03	0.00	–	–
Model 2						
Total effects	0.14	0.03	0.08	0.19	5.03	0.000
Direct effect	0.11	0.03	0.06	0.16	4.34	0.000
Total indirect effect	0.04	0.02	0.01	0.07	–	–
Indirect effect (X→M1→Y)	0.01	0.01	0.00	0.03	–	–
Indirect effect (X→M2→Y)	0.02	0.01	0.00	0.05	–	–
Indirect effect (X→M1→M2→Y)	0.01	0.01	0.00	0.02	–	–

Similarly, for non-supportive parental responses, the serial mediation results also aligned with our expectations. The full model was significant, F(1, 408) = 25.27, *p* < 0.001, R^2^ = 0.06, and the indirect effect of non-supportive parental emotion responses on depression through the mediators ECBs and cognitive reappraisal was significant (see Table 6 and Fig. 4b). Non-supportive parental emotion responses positively predicted depression, negatively predicted ECBs, but did not predict cognitive reappraisal. Furthermore, cognitive reappraisal negatively predicted depression, and ECBs positively predicted cognitive reappraisal, while ECBs did not directly predict depression.

## Discussion

The current research aimed to explore the underlying processes through which parental emotion responses may exert an influence on mental health outcomes. Specifically, across two studies, we examined the extent to which individuals believe emotions can be controlled (ECBs) and cognitive reappraisal mediated the relationship between retrospective perceptions of supportive and non-supportive parental emotion responses and depression in adulthood. Single and serial mediational analyses were conducted to examine whether ECBs and cognitive reappraisal, independent of one another as well as acting together, were responsible for the influence of parental emotion responses on depression.

Across both studies, the association between recalled experiences of supportive parental emotion responses and depression was mediated by cognitive reappraisal, confirming our first hypothesis. This finding is consistent with prior research noting the importance of parenting constructs for the development of emotion regulation strategies and depression. For example, Betts et al.^21^ found that parenting characterised by low nurturance alongside low levels of cognitive reappraisal were associated with higher depressive symptomatology, although they did not test for mediation in their study. Moreover, in findings that are analogous to ours, Chen et al.^36^ reported that the relationship between parental attachment and depressive symptoms in adolescents was mediated by cognitive reappraisal. However, to our knowledge, an explicit test of the potential mediating role of cognitive reappraisal in the association between supportive parental emotion responses and depression until now remains unexplored. Our notions and findings are in line with emotion socialisation theory^7^ which proposes that one pathway of emotion socialisation consists of parent’s responses to, and discussions of, emotions. We posit that when a parent responds supportively to their child’s negative emotion, this allows the child to make sense of their emotional experience, thereby removing the need to avoid or escape these difficult feelings. This, in turn, provides them with opportunities to develop adaptive emotion regulation strategies, such as cognitive reappraisal, protecting their mental health. Our findings are based on retrospective reports of parental emotion responses, and we acknowledge the limitations associated with this approach (see *Limitations* below). Nevertheless, we cautiously suggest that our examination of the experiences of a broad age-range of adults imply that supportive parental emotion responses may have long-term impacts on psychological well-being through their effects on emotion regulation strategies. If consolidated, these findings could have important implications for the prevention of depression through coaching parents to provide more supportive emotion responses for their children. To develop these claims, we encourage longitudinal research to investigate the lasting impact of parental emotion responses on psychological development^37^.

Our studies showed notable differences with respect to non-supportive emotion responses. In Study 1, contrary to prior predictions, and in opposition to our first and third hypotheses, the relationship between non-supportive parental emotion responses and depression was not mediated by cognitive reappraisal, evidenced in both the simple and serial mediation models. However, in Study 2, with its larger sample size, this mediation pathway emerged as significant, with non-supportive parental emotion responses negatively predicting cognitive reappraisal, which in turn negatively predicted depression. This finding from our second study aligns with previous research suggesting that non-supportive parental emotion responses negatively impact children’s emotional development and are linked with decreased use of cognitive reappraisal^14,38^. One possible explanation for the discrepancy between our two studies could be due to the effect of non-supportive parental emotion responses being more subtle and requiring greater statistical power to detect. Although the association was not statistically significant in Study 1, the pattern of results was in the expected direction, suggesting that the effect may not have been detectable given Study 1’s limited power. Notably, the differences in effect sizes in both studies between supportive and non-supportive parental emotion responses on cognitive reappraisal suggest that these constructs are distinct rather than simply two sides of the same coin^39–41^. We speculate that while supportive responses enhance cognitive reappraisal, functioning as a protective factor against depression, non-supportive responses may have a more pronounced impact on other regulation strategies such as expressive suppression^22,42^. Hence, future research may wish to go beyond exploring cognitive reappraisal and consider a variety of emotion regulation strategies^43^.

With regard to ECBs, our studies also revealed important differences. In Study 1, neither the relationship between supportive nor non-supportive parental emotion responses and depression was mediated by ECBs, which contrasted with both our second and third hypotheses. However, Study 2, with its enhanced statistical power, found that ECBs did mediate these relationships. Specifically, supportive parental emotions positively predicted ECBs, which in turn negatively predicted depression. Conversely, non-supportive parental emotion responses negatively predicted ECBs, which in turn negatively predicted depression. In the pathways of our mediation analyses in Studies 1 and 2, ECBs were found to predict depressive symptoms, consistent with research elsewhere^28–30^.

Our third hypothesis proposed a serial mediation model whereby parental emotion responses would predict ECBs, which would then predict cognitive reappraisal, which would in turn predict depression. In Study 1, this serial mediation was not supported. However, Study 2 confirmed this hypothesised pathway for both supportive and non-supportive parental emotion responses. These findings suggest a developmental cascade where parenting practices influence children’s beliefs about emotion controllability, which subsequently shapes their adoption of adaptive emotion regulation strategies like cognitive reappraisal, ultimately affecting mental health outcomes. This model represents a novel contribution to the literature by integrating multiple theoretical perspectives on emotion socialisation, beliefs, regulation, and psychopathology.

The divergent findings between our two studies highlight several important methodological considerations. First, the larger sample size in Study 2 likely provided greater statistical power to detect the more subtle mediating effects of ECBs and the serial mediation pathways. This underscores the importance of adequately powered samples when investigating complex psychological processes. Second, the replication with consistent measures across both studies strengthens confidence in the findings where they converge, particularly regarding the mediating role of cognitive reappraisal in the relationship between supportive parental emotion responses and depression.

Variance in the findings between our two studies may also reflect demographic differences between the samples. Study 1’s sample diverged notably from UK population norms, with approximately half of the participants identifying as Asian (versus White), a younger average age (29 years), and more female participants (65.6%). In contrast, Study 2 featured a more representative sample with a balanced gender distribution (51.7%), higher average age (46 years), and an ethnic composition closer to the UK demographic profile^44^. These differences may have influenced our results in several ways. Research suggests females tend to perceive emotions as less controllable than males do, while older individuals similarly show a tendency to view emotions as less malleable^44^. Besides age and gender, cultural background is considered an important source of emotion controllability beliefs^45,46^. In many Asian contexts, emotions are often viewed as informational, subject to change, and to be accepted rather than controlled^47,48^— a perspective that differs from the Western emphasis on increasing positive emotions and decreasing negative emotions. This cultural variation could influence the relationships we observed, though we acknowledge that this notion is speculative since our data is based on ethnicity identification not current cultural identification. The sample composition of Study 2 may have provided a more balanced representation that allowed associations between parental responses, cognitive reappraisal, ECBs, and depression to emerge more clearly.

Although there are strengths to the current research, not least the novel underlying mechanisms that we investigated in relation to emotion socialisation and mental health, as well as the replication with a larger sample, we must also acknowledge some limitations. First, both studies relied on cross-sectional data. Mediation is a process that unfolds over time and is ideally tested using longitudinal designs. However, the use of cross-sectional mediation is common in psychological research and can be justified when there is theoretical or empirical support for the temporal ordering of variables^49^. It nonetheless carries the risk of bias. As such, any causal interpretations should be made with caution. We recommend future studies overcome these limitations by collecting longitudinal data and adding design features like experimental manipulation. Second, we explored only general beliefs about the controllability of emotions (e.g., “No matter how hard they try, people can’t really change the emotions they have”) rather than personal beliefs (e.g., “If I want to, I can change the emotions that I have”). While this aligns with common practice in the field^50^, personal beliefs may have distinct implications for psychological health, with some research suggesting stronger associations with well-being and psychological distress^30^. Therefore, we encourage future research to unpick these possibilities. Lastly, our reliance on retrospective reports of experiences of parental emotion response introduces potential reliability concerns. Adult’s memory of the family environment often diverges from ‘truth’, with individuals over simplifying events that occurred years prior, leading to the presence of false negatives, measurement error, and biases in the evidence reviewed^51,52^. While these perspectives and memories may be pertinent for development^53^, crucially for our hypotheses, memories are often reconstructed to align with current outcomes. Hence, participants’ current mental health may have shaped their views of earlier experiences of parental emotion socialisation and vice versa^54^. While research suggests these biases aren’t sufficient to invalidate retrospective measures^52^, future studies would benefit from observational assessments to enhance reliability.

## Conclusions

In sum, our research across two studies provides evidence for the importance of both cognitive reappraisal and emotion controllability beliefs in mediating the relationship between parental emotion response and depression. While Study 1 identified cognitive reappraisal as a mediator only for supportive parental responses, Study 2 provided support for its role for both supportive and non-supportive responses and additionally established ECBs as an important mediator in these relationships. The serial mediation findings from Study 2 further suggest a possible developmental cascade whereby parental emotion responses shape emotion beliefs, which influence cognitive reappraisal, ultimately affecting mental health outcomes. This highlights the importance of emotion controllability beliefs in shaping both cognitive reappraisal and depression. These findings have important implications for prevention and intervention efforts targeting depression through strategies aimed at enhancing supportive parental emotion responses and developing adaptive emotion beliefs and regulation strategies. We encourage future research to build upon these findings using longitudinal designs and diverse samples to further elucidate these complex relationships.

## Methods

Both studies were approved by the Research Ethics Panel of University College London and fulfilled the ethical standard procedures recommended by the British Psychological Society. All participants gave informed consent to participate in the study.

### Study 1

#### Participants

Participants were recruited using convenience sampling, drawing on a research participation pool and online social media platforms. Two-hundred-and-twenty-seven participants were recruited, 11 of whom were excluded from the analysis due to response speeding (i.e., survey completion in under 10 min) and one for not meeting the age inclusion criteria ( ≥ 16). The final sample comprised 215 adults and adolescents (n = 141 females, n = 69 males, n = 1 non-binary) over the age of 16 (*M* = 29.10; *SD* = 10.14; Range = 18 to 81). For levels of depression, see Table 7. 43% of participants in this sample self-identified as ‘White’ (n = 91), 1% as belonging to ‘multiple ethnic groups’ (n = 3), 48% as ‘Asian or Asian British’ (n = 101), 1% as ‘Black or Black British’ (n = 3), and 6% identified as ‘other ethnic group’ (n = 12). Compared with UK census data, the present sample consisted of a disproportionally larger percentage of young, female, and Asian participants^55^. The sample achieved statistical power of 0.99 to detect medium effect sizes^56^.

**Table 7:** Prevalence levels of depression.

Levels of depression	% (n)
Typical	55.0% (n = 116)
Mild	15.6% (n = 33)
Moderate	22.3% (n = 47)
Severe	2.8% (n = 6)
Extremely severe	4.3% (n = 9)

### Procedure

Using the online data-collection platform, Qualtrics, participants were provided with an information sheet and consent form, before being asked to complete demographic information, and the scales detailed below. The survey took on average approximately 20 min to complete.

### Measures

#### Parental emotion responses

The Coping with Children’s Negative Emotions Scale (CCNES)^57^ consists of nine scenarios (e.g. “When my parents see my becoming angry at a close friend, they usually…”) and six corresponding reactions (e.g., “…Get angry at me for losing my temper”). Scenarios and responses were reformatted as past-tense reflections for the purposes of retrospective reporting and measured on a 7-point Likert Scale from strongly disagree to strongly agree. Distress/punitive and emotion-focused/problem-focused subscales demonstrated high correlations (distress/punitive = 0.75, *p* < 0.001; emotion-focused/problem focused = 0.87, *p* < 0.001), and were merged into supportive and non-supportive parental-emotion-response composites (see, Mirabile et al.^58^; Morelen et al.^40^), with excellent internal consistencies (supportive α = 0.95 and non-supportive α = 0.92, respectively).

#### Cognitive reappraisal

Cognitive reappraisal was measured using the six-item subscale from the self-report Emotion Regulation Questionnaire (ERQ)^19^. Example items include, “I control my emotions by changing the way I think about the situation I’m in” and, “When I want to feel less negative emotion I change the way I’m thinking about the situation”. Items were rated on a 7-point Likert scale from strongly disagree to strongly agree. High scores reflected higher levels of cognitive appraisal. The ERQ had good internal consistency (α = 0.81).

#### Emotion controllability beliefs

The Theories of Emotions Scale^24^ included four items assessing ECBs (e.g., “Everyone can learn to control their emotions”). This was originally measured on a 6-point Likert scale but in the present study was measured on a 7-point Likert Scale (strongly disagree to strongly agree) to be consistent with the other emotion measures in the study. Higher scores reflect greater belief that emotions are controllable.

#### Depression

The depression subscale of the Depression, Anxiety, and Stress Scale (DASS-21) ^59^ was used, consisting of seven items (e.g., “I felt that I had nothing to look forward to”) rated on a 4-point-Likert scale. The participant indicated how often in the past week the statement applied to them, from never to almost always. Higher scores reflected higher levels of depression.

### Data analysis

Missing data was determined to be missing completely at random and accordingly remained in the data set. A univariate outlier analysis was conducted for all variables; four points laying outside of three standard deviations from the mean were identified and removed. The following assumptions for multivariate analysis were met: *linearity* was assessed by visual inspections of the scatterplots of each predictor variable with the outcome variable, *normality* was assessed via the central limit theorem (CLT) and through the inspection of P-P plots, *homoscedasticity* was assessed through the Durbin-Watson statistic. All analyses were conducted with SPSS (Version 28), and mediation analyses used the PROCESS macro (Version 4.1), a computational tool applied for observed variable path analysis such as mediation analysis^60^. We used 5000 bootstrap samples to examine the relationship between parental reactions and depression, mediated by ECBs and cognitive reappraisal. Confidence intervals were derived from this distribution to test for significance of indirect effects.

### Study 2

#### Participants

Participants were recruited through the online platform Prolific. Prolific uses stratified sampling, based on key demographic variables (age, sex and ethnicity) to align with the UK Office of National Statistics (ONS) data^55^. A total of four-hundred-and-twenty-three individuals were initially recruited; however, 8 participants were excluded for failing the attention check (i.e., responding to a fictional item that has a single correct response option), an additional 2 were removed for not completing any questions beyond the consent form, and a further 3 were removed due to missing data. The final sample comprised 410 adults and adolescents (n = 212 females, n = 197 males, n = 1 non-binary) over the age of 18 (M = 46.24; SD = 15.38; Range = 18 to 79). For levels of depression, see Table 8. 85% of participants in this sample self-identified as ‘White’ (n = 350), 2% as belonging to ‘multiple ethnic groups’ (n = 7), 8% as ‘Asian or Asian British’ (n = 32), 3% as ‘Black or Black British’ (n = 12), 2% identified as ‘other ethnic group’ (n = 8), and 0% chose not to say (n = 1).

**Table 8:** Prevalence levels of depression.

Levels of depression	% (n)
Typical	48.5% (n = 199)
Mild	12.2% (n = 50)
Moderate	21.7% (n = 89)
Severe	8.3% (n = 34)
Extremely severe	9.3% (n = 38)

#### Procedure and measures

The procedure, design, and measures were the same as in Study 1, aside from the recruitment process, which is described above.

#### Data analysis

Missing data was determined to be missing completely at random and accordingly remained in the data set. Linearity was assessed by visual inspections of the scatterplots of each predictor variable with the outcome variable, normality was assessed via the central limit theorem (CLT) and through the inspection of P-P plots, and homoscedasticity was assessed through the Durbin-Watson statistic. All analyses were conducted with SPSS (Version 29), and mediation analyses used the PROCESS macro (Version 4.2), a computational tool applied for observed variable path analysis such as mediation analysis^60^. We used 5,000 bootstrap samples to examine the relationship between parental reactions and depression, mediated by ECBs and cognitive reappraisal. Confidence intervals were derived from this distribution to test for significance of indirect effects.

## Data Availability

The data that supports the findings of this study are available from the corresponding author, SP, upon reasonable request.
